# Polishing of 4Y- and 5Y- zirconia: effects on roughness, color and gloss

**DOI:** 10.1007/s00784-025-06244-1

**Published:** 2025-02-24

**Authors:** Laura Koch, Sebastian Hahnel, Angelika Rauch, Martin Rosentritt

**Affiliations:** Department of Prosthetic Dentistry, UKR University Hospital Regensburg, 93042 Regensburg, Germany

**Keywords:** Zirconia, 4Y-TZP, 5V-TZP, Polishing, Roughness, Colour, Gloss

## Abstract

**Objectives:**

To investigate the effects of polishing systems on the roughness, gloss and color of two zirconia materials as a function of the rotational speed and number of polishing movements.

**Materials and methods:**

Specimens (*n* = 8/group) from 4Y-TZP and 5Y-TZP were milled, diamond grinded and polished. The intraoral polishing varied in the number of polishing steps, the grain size and the matrix. Different number of polishing movements and rotation speed were used. Roughness (ISO25178-2:2021), gloss (ISO2813:2014) and color stability (ISO/CIE 11664–4:2019) were determined. Statistics: Shapiro–Wilk, one-way analysis, Bonferroni, Pearson, variance analysis/intermediate sub-effects (α = 0.05).

**Results:**

Roughness Sa varied between 2.45 ± 0.93 µm and 6.47 ± 0.26 µm (4Y-TZP) and 2.31 ± 0.19 µm and 6.54 ± 0.22 µm (5Y-TZP). Maximum roughness Sz ranged from 32.92 ± 12.59 µm to 99.32 ± 19.87 µm (4Y-TZP) and 31.45 ± 3.02 µm to 90.75 ± 12.59 µm (5Y-TZP). Different gloss from 28.9 ± 4. 23 to 102.39 ± 18.63 GU (4Y-TZP) and 33.19 ± 3.68 to 101.28 ± 10.00 GU (5Y-TZP) was found. ΔE results ranged from 0.39 ± 0.34 to 6.30 ± 1.22 (4Y-TZP) and 0.87 ± 0.71 to 7.50 ± 1.67 (5Y-TZP). Significant (p ≥ 0.095) intermediate sub-effects were found.

**Conclusions:**

Polishing led to a reduction in roughness, an increase in gloss and a significant change in color. Polishing had a stronger effect on 5Y-TZP.

**Clinical relevance:**

The correct use of the polisher type (stages, binder) and its specific application (rotational speed, movements) can improve roughness, gloss and color variations.

## Introduction

Monolithic zirconia restorations are increasingly becoming the focus of interest due to their high strength and efficient fabrication mode. Monolithic processing enables minimal invasive preparation and minimizes the risk of chipping, delamination and fractures. [1–3] Zirconia with yttria concentrations of 4 mol% or 5 mol% (4Y- or 5Y-TZP) shows an increased translucency and is therefore an aesthetic alternative to standard veneered 3Y-TZP. [4, 5] However, even with fully monolithic zirconia, the restoration must be carefully adjusted to achieve an optimal occlusal and proximal contact situation. The adaptation is usually carried with a diamond bur under water cooling, leading to roughening of the surface. Therefore, final polishing is mandatory [2, 6–10].

The efficacy of the polishing procedure can be verified by measurements of surface roughness, gloss, and color [6]. It is assumed that dental restorations with a surface roughness exceeding a threshold value (Profiliometric Ra = 0.2 µm) are more susceptible to the accumulation of plaque, which can lead to (secondary) caries, gingivitis and periodontal disease. [11–13] Such rough surfaces may affect the patient’s comfort, as unevenness in the size range of 0.25–0.5 µm can already be detected by the tongue [14]. Smooth and polished surfaces enhance the natural appearance of a dental restoration by increasing gloss and may thus improve the aesthetic appearance. [6, 10] Polishing also prevents excessive wear of the antagonistic tooth [8, 12, 13, 15] and enhances flexural strength. [16, 17] In addition, smooth surfaces are subject to less wear and are less susceptible to hydrothermal degradation, thus extending the service life of a restoration. [7, 8, 18, 19] Sz captures the variations between the valleys and reflects the more extreme surface features. Deep damage in particular, i.e. correspondingly high SC values, can be regarded as the cause of altered reflection and refraction of light, which can therefore influence the translucency, gloss and color behavior and thus the esthetics of the restoration. Finishing and polishing also affect the color properties and translucency of monolithic zirconia. [10, 20–23]

Polishing systems differ in terms of the number of polishing steps to be applied, the type of abrasive particles (diamond, silicon dioxide, aluminum oxide), the concentration of abrasive particles and the bonding agent used (natural rubber, polyurethane). [24–26] Nevertheless, the restorative surface quality is relevantly improved by polishing, which is regardless of the individual polishing set applied. [13, 27–29] However, the number of polishing steps and speed are important for both gloss and roughness [6]. It has been reported that there is no difference in roughness in polished zirconia after treatment with two or three step polishing systems. [30] However, three-stage polishing regimes seemed to produce a tendency towards lower roughness values compared to the two-stage process. [31] Producing a smooth surface always depends on the sequential application of all polishing steps, with each progressive step leading to an improvement in gloss and a reduction in roughness. [6] It was shown that polishing at a higher speed (15,000 vs 5,000 rpm) produces higher gloss and lower roughness. [6] The maximum speed of 20,000 rpm recommended by the manufacturers produces a smoother surface, but also a significant increase in temperature. [32] To prevent the accumulation of excessive heat, it might be advisable to polish longer but with low speed or, alternatively, to polish with high speed and use cooling intervals [32].

Differences in the type of diamond particles (natural or synthetic), shape, grain size, density and binder material can lead to different polishing results. [29] Diamond polishing systems were more effective than silicon carbide systems in reducing the surface roughness of zirconia, which can be attributed to the higher hardness of the diamonds. [29] The polishing results might also be affected by the embedding medium, as the cutting performance depends on the particles remaining on the polisher. [6] As polishing is a manual work step, it is dependent on the individual contact pressure, the freely selectable speed, the speed and the number of movements performed [24] as well as the material properties such as hardness, strength or modulus of elasticity. [13]

The aim of this study was to investigate the effect of polishing on roughness, gloss and color of two zirconia materials in dependence on speed and required number of grinding processes of 2- or 3-stage polishing systems with different diamond particle bonding regimes. The null hypothesis was that neither the type of polisher used nor the processing conditions influence roughness, gloss and color of the two zirconia materials.

## Material and methods

**Fig. 1:**
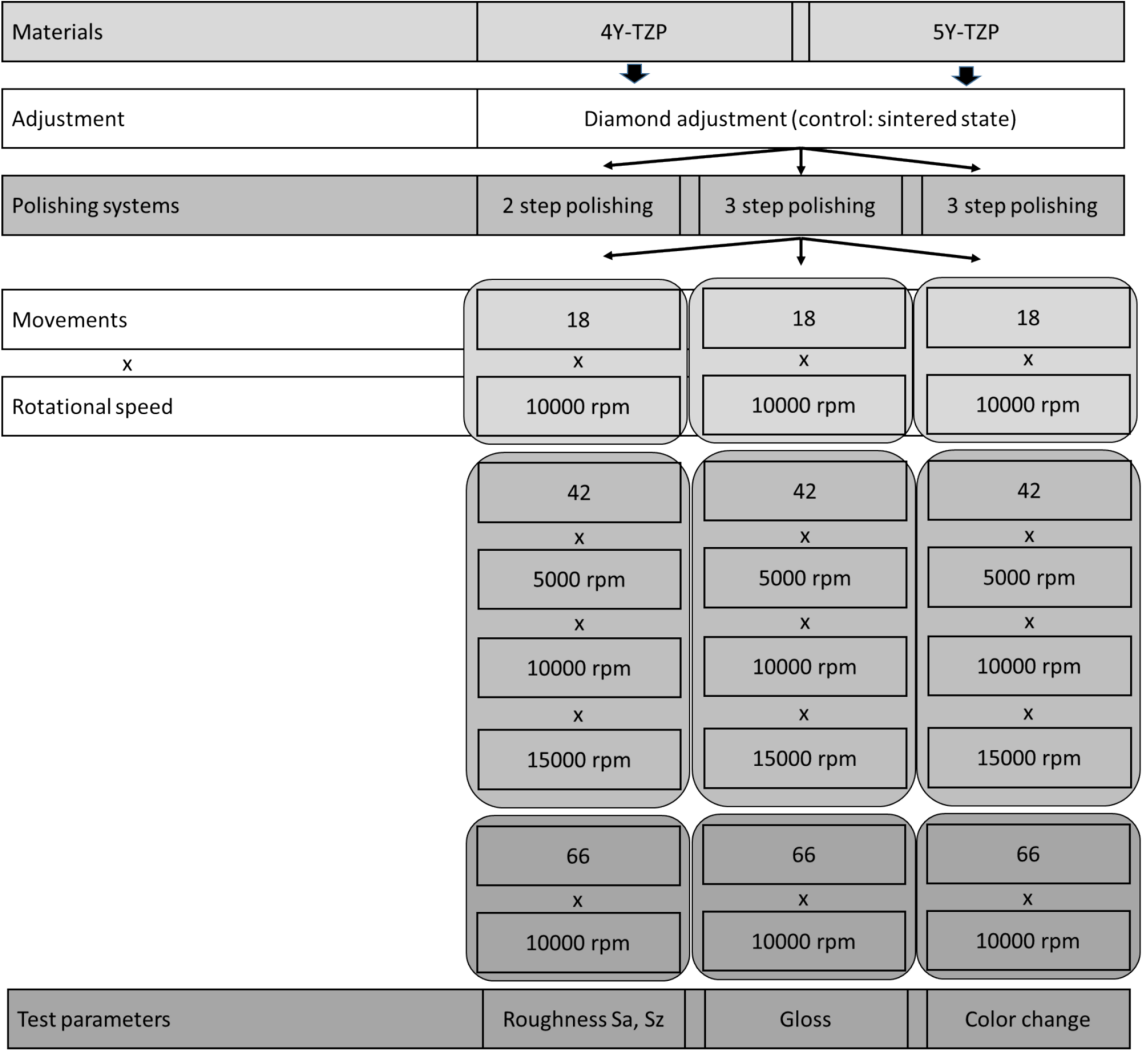
Study design

Standardized specimens (*n* = 8 per group, 5.9 mm diameter, 3 mm height) were milled (inLAB MC X5, Sirona Dentsply, G; settings: detail level very high, machining mode normal) from 4Y-TZP (DD cube ONE, G824, Dental Direkt, G; strength 1200 ± 150 MPa) and 5Y-TZP (DD cubeX^2^, G714, Dental Direkt, G) and sintered (inFire HTC speed, Sirona Dentsply, G) (Fig. 1; Table 1). The number of disk-shaped samples was 128 per material (120 polished, 8 as a sintered control) divided into 15 groups.

**Table 1:** Materials and methods

Materials	4Y-TZP (DD cube ONE, G824, Dental Direkt GmbH, G)		5Y-TZP (DD cubeX^2^, G714, Dental Direkt GmbH, G)
Flexural strength MPa Fracture toughness MPa√m Composition	1200 ± 150 > 10 ZrO₂ + HfO₂ + Y₂O₃ ≥ 99.0 Y₂O₃ < 8.0 Al₂O₃ < 0.15 Other oxides < 1.0		700 ± 100 4.0 ZrO₂ + HfO₂ + Y₂O₃ ≥ 99.0 Y₂O₃ ≤ 10.0 Al₂O₃ ≤ 0.01 Other oxides < 1.0
Pretreatment/adjustment	882-014F-FG, grain 27 to 76 µm, NTI, G		
Polishing (all NTI, G, Matrix)	Cera Glaze (natural rubber, 3 step)	CreaShine Trio (polyurethane, 3 step)	CeraShine Duo (natural rubber, 2 step)
step 1 (grain 107–181 µm)	(P335) 802 204 030 533 060	(PT2620) 803 204 030 533 060	–
step 1 (grain 64–126 µm)	–	–	(PD2630) 804 204 030 524 060
step 2 (grain 64–126 µm)	(P3035) 802 204 030 523 060	(PT2630) 803 204 030 523 060	–
step 2 (grain 27–76 µm)	–	–	(PD2640) 804 204 030 514 060
step 3 (grain 27–76 µm)	(P30035) 802 204 030 513 060	(PT2640) 803 204 030 513 060	–
Movements (with 10,000 rpm) x rotational speed (with 42 movements)	18 × 10,000	42 × 5,000 42 × 10,000 42 × 15,000	66 × 10,000
Test parameters	Roughness Sa, Sz (ISO 25178–2:2021)	Gloss (ISO 2813:2014)	Changes in color (ΔE; ISO/CIE 11664–4:2019)

**Fig. 2:**
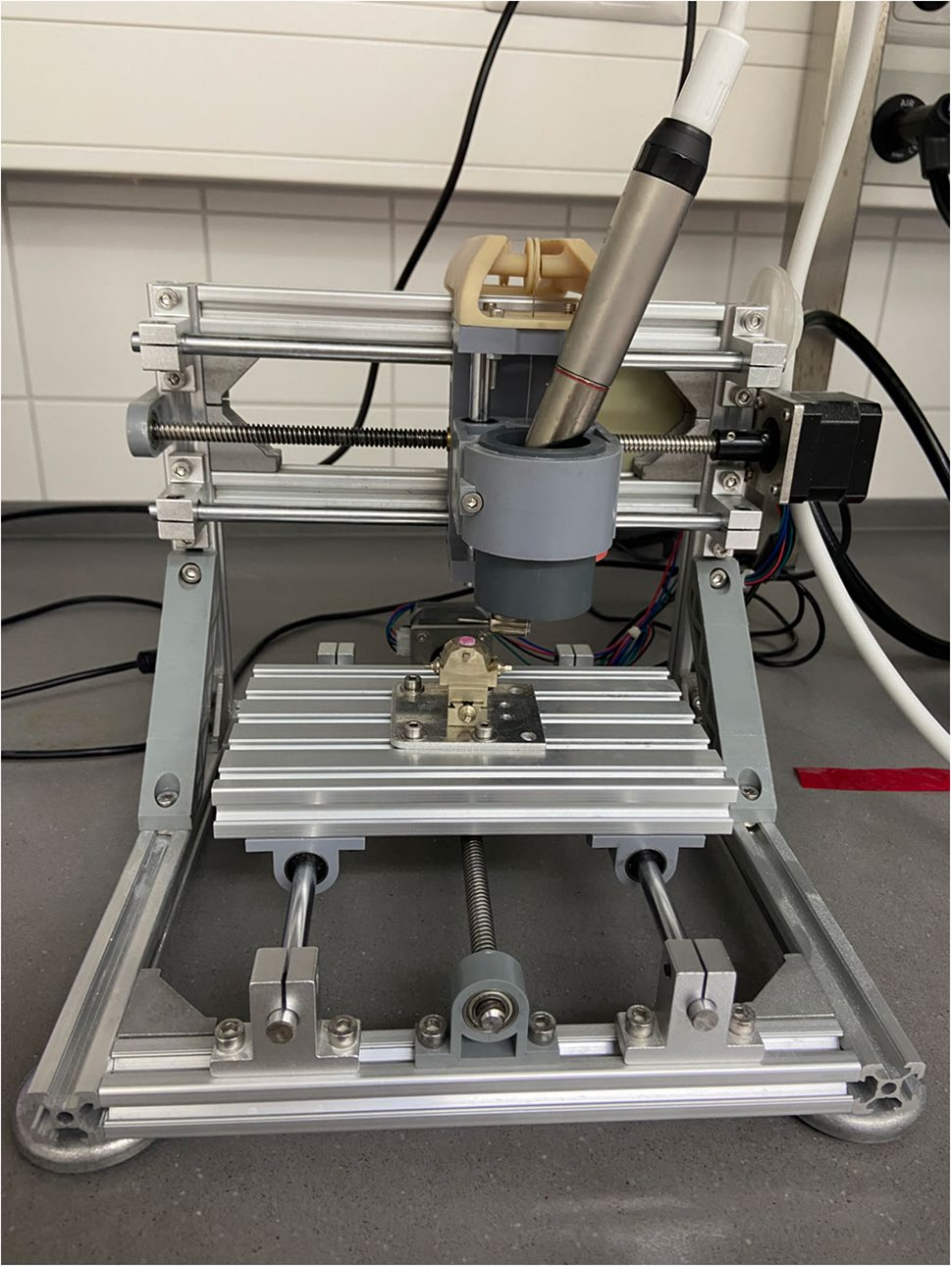
Device for standardized polishing

As a pre-treatment for the subsequent different polishes, all specimens were prepared using a contra-angle handpiece (T1 line, Sirona) by grinding with a diamond bur (882-014F-FG, 27 to 76 µm, NTI, Kahla, G) under standardized conditions (water cooling at a rate of 50 ml/min, 2 N, 40,000 rpm, 18-fold forward and backward movement at a constant speed of 500 mm/min). To guarantee consistent test conditions all grinding and polishing was performed with a special two axis motor controlled polisher test bench (Fig. 2).

The specimens were processed using three different intraoral polishing systems which differed in the number of polishing steps (2 × vs. 3x), grain size (grain: 107–181 µm vs. 64–126 µm vs. 27–76 µm) and matrix (natural rubber, polyurethane; Table 1). Different application parameters (time of application and rotation speed) of the contra-angle handpiece (T1 line, Sirona) were simulated by changing the number of movements (forward and backward: 18x, 42x, 66 × at 10,000 rpm) and adapting rotational speed (5,000, 10,000, 15,000 rpm at 42x). All specimens were polished with a constant speed of 500 mm/min with 2 N and permanent water cooling at a rate of 50 ml/min. After each polishing step, the polishing direction was changed by 90° to the grinding direction. Eight specimens of each material were retained in the sintered state as a control.

Roughness (Sz, Sa) was determined using a 3D laser scanning microscope (KJ 3D, Keyence, Osaka, Japan; scanning area 2000 × 1000 µm, λC = 0.08 mm) in line with the ISO 25178–2:2021 standard. [33] Gloss was measured using a gloss meter (ZGM, Zehntner Testing, Sissach, Switzerland) at an angle of 60° in accordance with ISO 2813:2014. [34] The color stability was determined with a spectrophotometer (CM-3500d, Konica-Minolta, Chiyoda, Japan) against black background and assessed using the CIELAB system as per ISO/CIE 11664–4:2019. [35] Changes in color (ΔE) were calculated.

Statistical analysis was performed using SPSS 28.0 (IBM, Armonk, NY, USA). Normal distribution of the data was confirmed using the Shapiro–Wilk test. Means and standard deviations were calculated and analyzed using one-way analysis of variance and Bonferroni test for post-hoc analysis in comparison to the sintered control. Pearson correlation (PC) was determined for each parameter. Univariate variance analysis was used to represent the intermediate sub-effects. The significance level was set to α = 0.05.

## Results

### 4Y-TZP

**Fig. 3:**
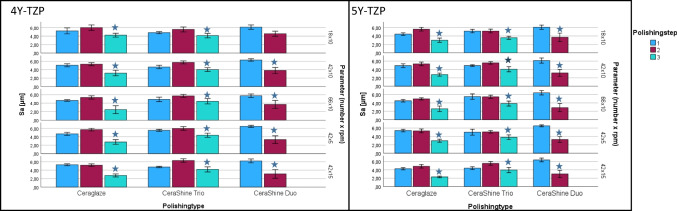
Mean Sa [µm] for the two zirconia materials (left: 4Y-TZP, right: 5Y-TZP; mean ± standard deviation [µm]. Depending on polisher types (Ceraglaze, CeraShine Trio, CeraShine Duo), number of polishing steps (1, 2, 3) and parameters (movements [18, 42, 66] x rotational speed [5: 5000 rpm-10: 10,000 rpm −15: 15,000 rpm]. Stars indicate significant differences to the sintered control 4Y-TZP: 5.59 ± 0.55 µm; 5Y-TZP: 5.06 ± 0.41 µm

Sa: Mean roughness Sa varied between 2.45 ± 0.93 µm and 6.47 ± 0.26 µm with significant (*p* < 0.001) differences between the results. Data and individual differences were displayed (Fig. 3). Sa of the sintered control was 5.59 ± 0.55 µm.

**Fig. 4:**
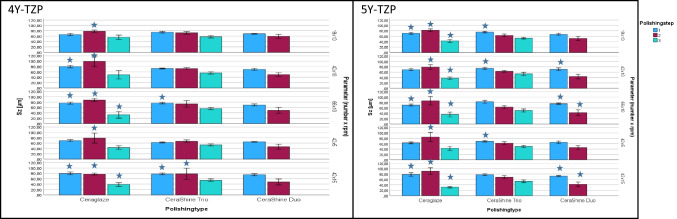
Mean Sz [µm] for the two zirconia materials (left: 4Y-TZP, right: 5Y-TZP; mean ± standard deviation [µm]. Depending on polisher types (Ceraglaze, CeraShine Trio, CeraShine Duo), number of polishing steps (1, 2, 3) and parameters (movements [18, 42, 66] x rotational speed [5: 5000 rpm-10: 10,000 rpm −15: 15,000 rpm]. Stars indicate significant differences to the sintered control 4Y-TZP: 58.78 ± 2.42 µm; 5Y-TZP: 56.28 ± 2.69 µm

Sz: Mean maximum roughness Sz varied between 32.92 ± 12.59 µm and 99.32 ± 19.87 µm with significant (*p* < 0.001) differences between the results. Individual differences were found in comparison to the control (Fig. 4). The roughness Sz of the sintered control was 58.78 ± 2.42 µm.

**Fig. 5:**
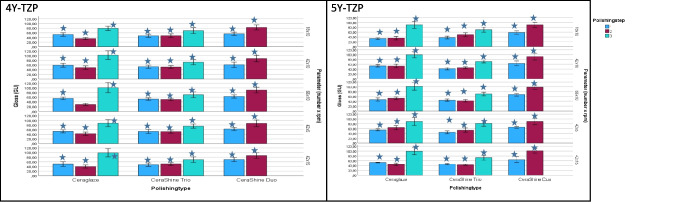
Mean Gloss [GU] for the two zirconia materials (left: 4Y-TZP, right: 5Y-TZP; mean ± standard deviation [µm]. Depending on polisher types (Ceraglaze, CeraShine Trio, CeraShine Duo), number of polishing steps (1, 2, 3) and parameters (movements [18, 42, 66] x rotational speed [5: 5000 rpm-10: 10,000 rpm −15: 15,000 rpm]. Stars indicate significant differences to the sintered control 4Y-TZP: 10.51 ± 2.43 GU; 5Y-TZP: 16.01 ± 3.74 GU

Gloss: Gloss values between 28.9 ± 4.23 and 102.39 ± 18.63 GU were found with significant (*p* < 0.001) differences between the data. Data and individual differences to the control (10.51 ± 2.43 GU) are shown (Fig. 5).

**Fig. 6:**
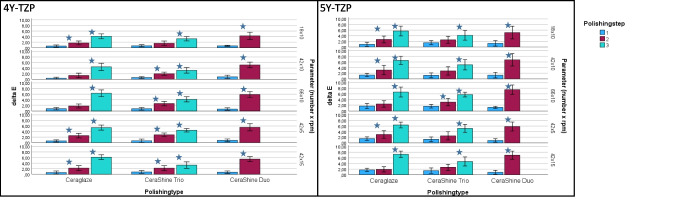
Mean ΔE [] for the two zirconia materials (left: 4Y-TZP, right: 5Y-TZP; mean ± standard deviation [µm]. Depending on polisher types (Ceraglaze, CeraShine Trio, CeraShine Duo), number of polishing steps (1, 2, 3) and parameters (movements [18, 42, 66] x rotational speed [5: 5000 rpm-10: 10,000 rpm −15: 15,000 rpm]. Stars indicate significant differences between the individual polishing steps

Color: ΔE results verified significantly (*p* < 0.001) between 0.39 ± 0.34 and 6.30 ± 1.22. Individual significant differences were found between the individual polishing steps (Fig. 6).

Sa showed significant correlations to Sz (Pearson 0.708; *p* < 0.001), gloss (−0.559; *p* < 0.001) and ΔE (−0.686; *p* < 0.001). Sz provided significant correlations to gloss (−0.645; *p* < 0.001) and ΔE (−0.741; *p* < 0.001). For gloss significant correlations were found with ΔE (0.685; *p* < 0.001). For the polishing type no correlations (−0.791/0.560; *p* ≥ 0.058) were found. The polishing steps provided correlations to all tested parameters (−0.791/0.763; *p* < 0.001). The polishing parameters (number x rpm) had a correlation to gloss (0.207; *p* < 0.001) and ΔE (0.113; *p* = 0.027).

### 5Y-TZP

Sa: Mean roughness Sa varied between 2.31 ± 0.19 µm and 6.54 ± 0.22 µm with significant (*p* < 0.001) differences between the results. Data and individual differences were displayed (Fig. 3). Sa of the sintered control was 5.06 ± 0.41 µm.

Sz: Mean maximum roughness Sz varied between 31.45 ± 3.02 µm and 90.75 ± 12.59 µm with significant (*p* < 0.001) differences between the results. Individual differences were found in comparison to the control (Fig. 4). The roughness Sz of the sintered control was 56.28 ± 2.69 µm.

Gloss: Gloss values between 33.19 ± 3.68 and 101.28 ± 10.00 GU were found with significant (*p* < 0.001) differences between the data. Data and individual differences to the control (16.01 ± 3.74 GU) are shown (Fig. 5).

Color: ΔE results verified significantly (*p* < 0.001) between 0.87 ± 0.71 and 7.50 ± 1.67. Individual significant differences were found between the individual polishing steps (Fig. 6).

### Correlations

Sa showed significant correlations to Sz (Pearson 0.725; *p* < 0.001), gloss (−0.570; *p* < 0.001) and ΔE (−0.767; *p* < 0.001). Sz provided significant correlations to gloss (−0.676; *p* < 0.001) and ΔE (−0.743; *p* < 0.001). For gloss significant correlations were found ΔE (0.724; *p* < 0.001). The polishing type showed a correlation only for GU (−0.114; 0.023), but not for the other parameters (−0.097/0.053; *p* ≥ 0.302). Polishing steps correlated to all tested parameters (−0.558/0.646; *p* ≤ 0.001). The polishing parameters (number x rpm) correlated with gloss (0.224; *p* < 0.001), but not with the other parameters (−0.035/0.050; *p* ≤ 0.328).

### Intermediate sub-effects

Significant intermediate sub-effects (Table 2) were found on Sa (*p* = 0.744), Sz (*p* = 0.983), and ΔE (*p* = 0.811) for material*polishing type. ΔE was further influenced by polishing type*movements (*p* = 0.214), material*polishing type*polishing step (*p* = 0.095), material*polishing type*movements (*p* = 0.799), material*polishing step*movements (*p* = 0.752) and material*polishing type*polishing step*movements (*p* = 0.752). Gloss was influenced by material (*p* = 0.055), material*movements (*p* = 0.343), polishing step*movements (*p* = 0.163), material*polishing type*movements (*p* = 0.213) and material*polishing step*movements (*p* = 0.280).

**Table 2:** Intermediate sub-effects (*p*-values)

	Sa	Sz	Gloss	Delta E
Material	< .001	..016	.055	< .001
Polishingtype	< .001	< .001	< .001	< .001
Polishing Steps	< .001	< .001	< .001	< .001
Parameter (number x rpm)	< .001	< .001	< .001	< .001
Material * Polishingtype	.744	.983	< .001	.811
Material * Polishing Steps	< .001	.001	< .001	.015
Material * Movements	.011	.011	.343	.032
Polishingtype * Polishing Steps	< .001	< .001	< .001	< .001
Polishingtype * Movements	< .001	< .001	< .001	.214
Polishing Steps * Movements	.003	< .001	.163	.006
Material * Polishingtype * Polishing Steps	.031	< .001	.002	.095
Material * Polishingtype * Movements	< .001	< .001	.213	.799
Material * Polishing Steps * Movements	.024	.029	.280	.752
Polishingtype * Polishing Steps * Movements	< .001	< .001	.005	.011
Material * Polishingtype * Polishing Steps * Movements	.034	.015	.004	.752
R2	.839	.815	.866	.813

A multivariate linear model showed significant effects for material (*p* = 0.060) and movements (*p* = 0.176), but not for polishing steps and polishing systems (*p* < 0.001).

## Discussion

Parts of the null hypothesis that neither the polisher type nor the processing conditions had any influence on the results on different zirconia had to be rejected.

### Roughness Sa/Sz

With all polishing systems almost always a significant reduction in roughness (Sa) was achieved after the final polishing stage. Compared to the CST and CSD polishing systems, the CG system produced the lowest roughness values (Sa and Sz) after the last polishing stage in almost all groups of both materials. Taking into account the different measurement conditions, this result confirmed the influence of polishing on surface roughness. [7, 27, 36, 37] Different results from other studies could be explained by deviations in the polishing system, the polishing protocol and the lack of water cooling. [6, 10] Surface damage in particular, i.e. correspondingly high Sa or Sz values, can influence reflection and refraction of light, influencing translucency, gloss and color behavior of the restoration. The statistical evaluation showed that the roughness had a clearly negative correlation with the gloss and color change. Individual effects of the investigated parameters could not be shown, but the combination of material*polishing type has an effect on the quality of the roughness. This means that the choice of polishers is decisive for the roughness achieved on the respective zirconium oxides.

Binder: The type of binder seems decisive for reducing roughness. After the sequential application of all polishing steps for both materials, the rubber-containing polishing system showed better results in reducing Sa and Sz than the polishing system containing a polyurethane-based binder. Already Huh et al. reported an influence of the binder on zirconia polishing: a 3-step polishing system with a silicone-based binder produced higher Ra values than corresponding polishing systems with a synthetic rubber binder. [25] In contrast to the current study, a comparison of two-stage polishing systems that differed in their embedding medium (polyurethane vs. silicone) revealed that the polyurethane-based product produced lower roughness. [6]

Steps: The last polishing step produced the lowest roughness (Sa and Sz) for all polishing systems and for both materials in each group. Previous studies have shown that there is a decrease in surface roughness after sequential grinding and polishing. [6, 38] In the current study, there was almost always a significant roughness reduction (Sa) compared to the sintered control. With the 3-step polishing systems (CG and CST), Sa and Sz (except for CST, 5Y) for both materials changed only minimally after the second polishing step or even increased compared to the first polishing step. The desired polishing effect could only be achieved with completion of the third polishing stage. Caglar et al. also reported no significant differences in surface roughness produced by two different 3-step zirconia polishing systems. [28] In a study by Scherrer et al., no significant difference in surface roughness was identified after polishing zirconia crowns with a 2-step or 3-step polishing kit. [31] In their study, Gaonkar et. al. evaluated the efficiency of two commercially available polishing systems in reducing the surface roughness of monolithic zirconia after clinical adaptation. In contrast to the current study, they reported that the 2-stage polishing set achieved lower surface roughness values than the 3-stage polishing system, but there was no statistically significant difference between the two polishing systems. [30]

Rotation speed: There was a dependency between roughness Sa (and Sz) and rotation speed: polishing at the highest speed tended to produce lowest Sa (but also highest Sz) for both types of zirconia. This observation confirms the results of a previous study reporting that polishing zirconia at 15,000 or 40,000 rpm resulted in lower roughness. [6]

Movements: There was a correlation between the number of movements and roughness (Sa). Polishing with the highest number of movements tended to produce lowest Sa for both 4Y-TZP and 5Y-TZP. In contrast, Huh et al. showed that no significant differences in surface roughness could be observed between the polishing times of 60 and 120 s. [25] In the current study, the polishing duration ranged between 18 and 66 movements with an almost four times longer polishing duration, which could serve as an explanation for the effect observed in the present study.

### Gloss

All polishing systems achieved a significant increase in gloss for both types of zirconia, which was in the range between 70 and 100 GU. For 4Y-TZP and almost all groups, highest gloss after the last polishing step was achieved using the CG polishing system, and for 5Y-TZP using the CG and CSD polishing systems. Polishing three zirconia types with a 2-step diamond-impregnated and polyurethane-based polishing system, Abdulmajeed reported a surface gloss that was comparable to that of the sintered reference. [10] As in previous studies, we identified a correlation between roughness and gloss. [10, 36] The statistical evaluation showed that the polishing type, steps and parameters provided correlations with gloss, thus demonstrating the influence of the parameters on the quality of the gloss. Gloss is not only dependent on the polished material, but also on its combination with other parameters such as movements, polishing steps and polishing type. This shows not only the influence of the material and the processing parameters, but also the influence of the processor.

Binder: The diamond binder played a relevant role for increasing gloss. The rubber-based polishing system provided better results for increasing gloss than the polyurethane-based polishing system after the sequential application of all polishing stages. Two two-stage, diamond-impregnated polishing systems, which differed in their binder (polyurethane vs. silicone), showed no difference in gloss after the last polishing stage. [6]

Steps: When comparing two- and three-stage polishers, differences were identified between the two types of zirconia. For 4Y-TZP, the three-stage polishing system (CG) achieved higher gloss values after the last polishing stage for almost all groups than the two-stage system (CSD), which has the same binding medium. Chavali et. al. also found that each step led to an improvement in gloss. [6] For 5Y-TZP, the three-stage polishing system (CG) caused similar gloss values to the two-stage system (CSD) for almost all groups after the last polishing stage.

Rotation speed: There was a dependency between gloss value and speed. Polishing at the highest speed tended to result in lowest gloss values for both 4Y and 5Y. In contrast, Chavali et al. reported that polishing zirconia at 15,000 rpm resulted in higher gloss values than at 5,000 or 40,000 rpm. [6]

Movements: There is a correlation between the gloss value and the number of movements. Polishing with the lowest number of movements tended to result in lowest gloss for both 4Y-TZP and 5Y-TZP. A higher number of movements generally increased gloss, but reached a maximum at 42 movements.

### Delta E

For both materials, CG and CSD polishing systems produced the highest color difference (ΔE > 3.7) after the last polishing stage. These results confirm earlier reports suggesting that clinical adaptation has an influence on the color stability of zirconia. [21, 39] For example, Li et al. highlighted that the smoothest polished surface showed the greatest color difference. [22] Differences to results of other studies could be due to the base color or later coloration of zirconia [21] or distinct sintering conditions. [39] With polishing, all samples became greener (-a), bluer (-b) and darker (-L). Kim et al. also observed a significant change in color, a shift in the CIE a* value towards green and a reduction in brightness with polishing [20]. The authors assume that surface treatments such as polishing reduce the light scattering of zirconia surfaces. This phenomenon leads to a reduction in the spectral reflectance after polishing or glazing, which in turn decreases brightness. [20] The color change only showed a correlation with the polishing steps. Individual effects of the investigated parameters could not be shown. However, ΔE was influenced by material*polishing type*polishing step*movements in different combinations. This shows the complexity of the influence of the processing in particular on the color change. Therefore, a possible effect of the polishing parameters should be taken into account, especially when selecting the color.

Binder: Specimens polished with CG showed a greater color difference than those polished with CST, which indicates that the binder has an influence on the color. Some of the color differences measured in the present study were higher than ΔE 1 (= clinically imperceptible) and ΔE values between 1 and 3.7 (= clinically acceptable). [40, 41]

Steps: For both types of zirconia, no difference could be identified in the measured color distances between the two- and three-stage polishing systems.

Rotation: No clear correlation between speed and the resulting color distance could be identified.

Movements: Processing with 18 or 42 movements resulted in the smallest color change of all levels, while the greatest color change was identified for 66 movements. There was a dependency between color distance and the number of movements. Polishing with the highest number of movements led to greater color changes for both 4Y-TZP and 5Y-TZP.

### Materials

Roughness: Polishing results for the two zirconia were not identical. For all three polishing systems, 5Y-TZP featured lower Sa than 4Y-TZP, which is in contrast to a study reporting that the Yttria content had no significant influence on surface roughness. [38] A study by Nomoto et al. also reported no significant difference in surface roughness between two zirconia types after polishing. [36] However, another study indicated a significant increase in surface roughness and defects after milling and grinding of materials with higher Yttria contents. Higher roughness may lead to an impaired surface quality of transparent materials after dental adaptation and intraoral polishing. [13] However, more translucent zirconia materials may feature higher surface roughness after polishing due to the larger grain size. [42]

Gloss: Gloss of different types of zirconia was influenced by the type of the polishing system applied. CG and CST polishing systems produced similar gloss for 4Y-TZP and 5Y-TZP, yet CSD produced higher gloss values for 5Y-TZP. This observation is in contrast to studies that reported similar gloss for different types of zirconia. [10, 36]

Delta E: Polishing of 5Y-TZP resulted in a greater color difference than polishing 4Y-TZP. Nevertheless, the clinically relevant threshold value of ΔE = 3.7 after the last polishing step was exceeded by each polishing system and in both materials. This difference might be due to the different microstructures and compositions of the two types of zirconia. Compared to other types of zirconia, translucent zirconia might have a higher proportion of aluminum oxide and a lower proportion of zirconia, which produces higher transparency but also a higher susceptibility to color changes. [43]

### Polishing steps

Roughness: Although the 2-step system produced higher Sa values than CG and CST polishing systems after the first polishing step, it caused lower Sa values than CST after the third polishing step. It might be assumed that a third polishing stage therefore might not be mandatory to achieve an effective reduction in the Sa value. A 4-step polishing system produced lowest Ra when polishing zirconia compared to 1- or 2-step polishing systems. [13] The two polishing systems with binders based on natural rubber produced good final roughness after completion of all polishing steps. Both polishing systems are therefore suitable for polishing 4Y-TZP and 5Y-TZP, yet the 2-stage polishing system might be more efficient. The 3-stage polishing system was only slightly superior to the 2-stage system for reducing Sa and Sz.

Gloss: The 3-stage polishing system (CG) was slightly superior to the 2-stage polishing system for improving gloss value of 4Y-TZP. For polishing 5Y-TZP, the 2-stage polishing system produced similar results as the 3-stage polishing system, which indicates that both polishing systems are equally suitable for polishing 4Y-TZP and 5Y-TZP.

Delta E: For both types of zirconia, CG and CSD had a similar effect on color changes and both systems exceeded the clinically relevant threshold value of ΔE = 3.7 after the last polishing step for both types of zirconia.

The results of the current investigation must be interpreted within the limitations of the study, which includes the application of uncolored zirconia materials from a single manufacturer. Rather than in a clinical setting, polishing was performed on two-dimensional plates and not on multi-dimensional dental restorations.

## Conclusion

Most polishing led to a reduction in roughness, an increase in gloss but also to a significant change in color, always in relation to the sintered state.

The rubber polishing system showed lower roughness values and increased gloss values for both materials, but also led to a greater change in color.

The three-stage polishing system showed a slight superiority only for the effect on roughness over the two-stage polishing system.

The highest rotational speed tended to result in the lowest roughness values for both types of zirconia, but also the lowest gloss values.

Polishing with a higher number of movements tended to lead to the reduced roughness, increased gloss, but caused higher color changes. An excessive increase in the number of grinding processes has hardly any effect.

The effects of the polish are only partially dependent on the material.

## Data Availability

No datasets were generated or analysed during the current study.
